# Pathological Findings of Canine Idiopathic Pericarditis and Pericardial Mesotheliomas: Correlation with Clinical and Survival Data

**DOI:** 10.3390/vetsci8080162

**Published:** 2021-08-10

**Authors:** Michela Levi, Federico Parenti, Luisa Vera Muscatello, Stefano Battaia, Roberto Santilli, Manuela Perego, Vincenzo Montinaro, Federico Massari, Giuseppe Sarli, Barbara Brunetti

**Affiliations:** 1 Department of Veterinary Medical Sciences, University of Bologna, Via Tolara di Sopra, 50, Ozzano dell’Emilia, 40064 Bologna, Italy; michelalevi3@gmail.com (M.L.); federico.parenti3@studio.unibo.it (F.P.); giuseppe.sarli@unibo.it (G.S.); b.brunetti@unibo.it (B.B.); 2 Ospedale Veterinario I Portoni Rossi, Via Roma 57, Zola Predosa, 40069 Bologna, Italy; stefano.battaia@gmail.com (S.B.); man.perego@gmail.com (M.P.); montinaro.vincenzo@alice.it (V.M.); federicomassari@me.com (F.M.); 3 Clinica Veterinaria Malpensa, Viale Marconi 27, Samarate, 21017 Varese, Italy; rasantilmedvet@gmail.com; 4 Department of Clinical Sciences, Cornell University, 930 Campus Road, Ithaca, NY 14853, USA; 5 DOCVET, Clinica Veterinaria Nervianese, Via Lampugnani 3, 20014 Nerviano, Italy

**Keywords:** canine idiopathic pericarditis, mesothelioma, immune cells, survival analysis, pericardiectomy

## Abstract

Idiopathic pericarditis (IP) and pericardial mesothelioma (PM) are causes of pericardial effusion in dogs. Pericardiectomy can be a definitive treatment in the case of idiopathic pericardial effusion or a short-term intervention for mesothelioma. The aim of the present study was to investigate which histopathologic parameters are correlated with clinical outcomes in a cohort of dogs that underwent pericardiectomy. The histopathological findings of 22 IPs and 5 PMs were compared with clinical and survival data and the immunohistochemical characterization of immune cells (CD3, CD79α, Iba1). In IP, the mesothelium was lost in 20 cases, reactive in 9, atypical in 3, and mesothelial papillary hyperplasia (MPH) was observed in 4 cases. Numerous macrophages were found in both IPs and PMs especially at the superficial layer of the pericardium. T lymphocytes were observed in mild to moderate numbers and were more numerous than B lymphocytes in both IPs and PMs. MPH was correlated with the quantity of lymphoplasmacytic infiltrate in the superficial layer, inversely related to the thickness of the pericardium, and associated with a longer overall survival. Pericardial fibrosis was present in 19 out of 22 IPs and in all mesotheliomas and was correlated with increased time from initial presentation and pericardiectomy and lymphoplasmacytic infiltrate in the deep zone. Pericardial thickness was correlated with the amount of lymphoplasmacytic and macrophagic infiltrate in the deep zone. Mesothelioma was associated with an increased number of pericardiocentesis procedures before pericardiectomy and with the presence of macrophages in the superficial pericardial layer, edema, fibrin, and hemorrhage. Disease-free interval and overall survival were significantly shorter in patients with mesothelioma compared with IP.

## 1. Introduction

Pericardial diseases comprise a wide spectrum of conditions of both infectious and non-infectious etiology. Despite this, the pericardium is considered the most neglected research topic in mesothelial membrane research [1].

The pericardium consists of an external sac of fibrous connective tissue, called fibrous pericardium and an internal, serous pericardium. The latter covers the internal surface of the fibrous pericardium and the heart [2]. Mesothelial cells form a monolayer lining the serosal cavity and play an important role as a protective non-adhesive surface. The mesothelium is also involved in transport of solutes and cells across serosal cavities, antigen presentation, inflammation and tissue repair, coagulation, fibrinolysis, and tumor cell adhesion [3,4]. Causes of pericarditis in humans include viral, bacterial, fungal, uremic, and neoplastic factors, as well as post-acute myocardial infarction, post-cardiac surgery, mediastinal irradiation, and consequences of systemic autoimmune diseases [5,6]. Poorly understood causes of pericardial effusion in dogs are idiopathic pericarditis (IPs) and neoplastic effusion [7,8,9]. Currently, dogs without any evident cause for effusion after echocardiography and histological examination are considered presumptive idiopathic [6]. Certain large- or giant-breed dogs including Golden Retriever, German Shepherd, Great Dane, Great Pyrenean, and Saint Bernard are considered predisposed to developing IP [8,10,11,12]. The major complications of pericarditis are cardiac tamponade with and without hemodynamic instability in the short term, constrictive pericarditis in the long term and death [13]. Thoracoscopy with creation of a pericardial window appears to be an effective treatment for palliation of clinical signs in patients with pericardial effusion whereas pericardiectomy is the only treatment for permanent constriction [5,14]. Thoracoscopic pericardiectomy and pericardioscopy of the pericardium and pleura are recommended in dogs with echocardiographic idiopathic pericardial effusion [7].

Mesothelioma is the most common primary pericardial tumor and is almost always incurable in both human and canine patients [5,15]. Surgical intervention with pericardiectomy in patients with recurrent IP is often successful in ameliorating clinical signs, whereas in dogs with pericardial mesothelioma (PM) there is typically a persistent, recurrent pleural effusion, and prognosis is poorer [16]. The histological diagnosis of mesothelioma is challenging and fraught with pitfalls, since criteria for differentiating neoplastic from reactive mesothelial proliferations are scarce especially in the presence of small biopsies, concurrent inflammation of the serosa and in the absence of macroscopic serosal mass lesions [17,18,19]. In literature regarding both humans and canines, there is still a paucity of outcome data regarding patients with idiopathic and neoplastic pericardial disease requiring surgical intervention [6,20]. Furthermore, a recent systematic review of the efficacy of treatment options for pericardial effusion in dogs concluded that there is still not sufficient evidence to recommend one treatment option over another [21].

Current understanding of the sequence of pathological events leading to clinical manifestations of pericardial effusion and pericarditis in dogs is incipient [6,7,10,12]. The pathophysiological response of the human pericardium to injury is characterized by intense inflammation with or without effusion, and the clinical syndrome of pericarditis; chronic pericarditis often leads to various degrees of fibrogenesis with pericardial thickening [6,22]. Histopathologic findings and immunohistochemical analysis of inflammation in canine IPs were initially described by Aronsohn and colleagues (1999) and Day and colleagues (2002), respectively. These authors described a stratification of the inflammatory infiltrate in visceral/superficial and fibrous/deep pericardial zones, a variable degree of fibroplasia and presence of vascular and mesothelial changes [10,12].

The identification of key morphological lesions in the pericardium and their correlation with clinical and surgical outcome can aid in a better understanding of IP and PM, in the design of novel interventions, and in the interpretation and management of complications; however, at present, studies investigating this topic are lacking.

The aim of the present study was to investigate which histopathologic parameters are correlated with the clinical presentation and outcome in a cohort of dogs that underwent pericardiectomy and were diagnosed with IP or PM.

## 2. Materials and Methods

Dogs that had confirmed recurrent pericardial effusion and underwent a thoracoscopic pericardial window procedure or subtotal pericardiectomy via thoracotomy at the “Clinica Veterinaria Malpensa” and the “Ospedale Veterinario I Portoni Rossi”, Italy, between 2013 and 2018, were included in this retrospective cohort study. Dogs were included based on a histological diagnosis of IP and PM, not clinically evident as a mass. Exclusion criteria were the following: dogs presenting cardiac/pericardial masses consistent with neoplasia other than mesothelioma, and dogs with pericarditis due to uremia and renal failure, infectious diseases, and presence of foreign bodies. These pericardial diseases were excluded performing urinalysis, hematobiochemical exams, bacteriological, ultrasound, and histological examination.

Data were retrieved from records of client-owned pets. The pets were treated and housed according to standard hospital protocols for management of client-owned pets and no tests or treatments were conducted for research purposes. The owners provided standard signed consent for the data and images to be used for publication; the tissue samples utilized in this study were the same used for diagnostic purposes.

Collected data included age, breed, spay status, type of surgery (i.e., thoracoscopic partial window pericardiectomy and subtotal/subphrenic pericardiectomy via thoracotomy). Times from initial presentation for pericardial effusion and pericardiectomy and the number of pericardiocentesis performed before pericardiectomy to alleviate clinical signs of cardiac tamponade were recorded. Animals were followed up for 24 months after surgery. Survival time was measured from the date of pericardiectomy. Follow-up data were obtained via telephone interview with general care veterinarians and owners and/or recheck examination to determine the disease-free interval (DFI; time from surgery to the development of clinically evident pleural effusion), the overall survival (OS; time from surgery to death caused by cardiac or extracardiac causes or end of follow-up), disease specific survival (DSS; time from surgery to death caused by euthanasia due to the development of pleural effusion and respiratory distress), and the cause of death (COD). The follow-ups may have ended for the following reasons: (a) death by euthanasia because of the development of pleural effusion and respiratory distress, (b) death by other causes, or (c) end of study (24 months).

Pericardia from three dogs with normal cardiovascular and respiratory functions, euthanized for causes unrelated to cardiovascular and respiratory disease, were collected during necropsy at the Department of Veterinary Sciences of the University of Bologna, for use as negative controls.

Pericardial samples were collected intraoperatively or at necropsy and fixed in 10% neutral buffered formalin. Following fixation, multiple representative sections (at least three, including the most thickened areas) were taken from each sample of pericardium, and from paraffin embedded specimens, consecutive sections (5 μm) were routinely stained with hematoxylin and eosin (H&E) and Masson’s trichrome for light microscopy.

Each slide was evaluated by two pathologists (BB and ML) who reached a consensus on morphologic diagnosis and scoring of each parameter at 100× magnification. Semiquantitative assessment of histopathological parameters was scored and assigned to a category showing an ordered progression in severity (0 = normal; 1 = mild; 2 = moderate; 3 = severe histopathologic change) and in accordance with the principles proposed by Gibson-Corley and colleagues (2013) [23]. This resulted in the generation of both parameter-specific scores and summative histopathologic scores used for statistical analysis.

Serosal pericardium composed of visceral/internal/cardiac layers and the fibrous pericardium were included in the semiquantitative evaluation. The pericardial pleura, which consists of mesothelium lining the pleural/parietal/external aspect of the pericardial sac and lamina propria and the adipose pericardial tissue were not included in the scoring, since findings at these sites in IP were always absent and the wide variation in the amount of adipose tissue in this area of the pericardium would invalidate a comparison between the cases.

Histopathological evaluation included the severity of fibrosis assessed on Masson’s trichrome-stained sections; changes affecting the mesothelium lining the visceral aspect of the pericardial leaflet including loss of mesothelial cells, simple hyperplasia/reactive, mesothelial papillary hyperplasia (MPH), atypical mesothelial proliferation, and mesothelioma as proposed in literature [17,24]; and presence of granulation tissue, hemorrhage, edema, and fibrin overlying the pericardium or in the lamina propria. Adipose pericardial tissue and the external layer of mesothelium, lining the pleural aspect of the pericardial sac, were not included in the scoring, since changes at these sites were absent or minimal.

Immune cell infiltrates were evaluated considering their severity, composition (i.e., neutrophilic intravascular/perivascular, lymphocytes, and macrophages) and distribution in the pericardial layers (visceral/superficial, fibrous/deep) [10,12]. As described by Day and colleagues (2002), the pericardial leaflet can be divided into two distinct zones, the visceral/superficial zone consisting of a band of loose connective tissue located in the lamina propria immediately beneath the mesothelium, and the fibrous/deep zone located in the middle of the pericardium, composed of more densely packed collagen admixed with small blood vessels. In pericardial mesotheliomas, the pattern of the neoplasm, according to the latest proposed classification [19], amount of mitosis in 10 high power fields (total area 2.37 mm^2^ for 10 high power fields), anisocytosis and anisokaryosis, and lymphatic invasion were all recorded.

The sum of the histopathologic scores of inflammation parameters was calculated for the correlation to clinical outcome as a continuous variable and split into three classes based on the quantile (1 = lower quantile, 2 = median quantile, 3 = upper quantile) subdividing the accumulated range.

Pericardial thickness was assessed by manual image analysis (Image J software, National Institute of Health, Bethesda, MD, USA) with orthogonal measure perpendicularly to parallel tangents to the inner/cardiac pericardial surface and the edge of the deep zone of connective tissue, excluding the adipose pericardial tissue and the outer/pleural serous pericardial surface. The three most thickened areas present in the H&E slides were measured, avoiding areas with artifactual folding and/or tangential sectioning of the pericardial tissue in the paraffin block.

Pericardial thickness was examined for correlation to clinical outcome as a continuous variable and split into three classes based on the quantile (1 = lower quantile, 2 = median quantile, 3 = upper quantile) for statistical evaluation as a categorical variable.

Immunohistochemistry was performed using a commercial streptavidin–biotin–peroxidase technique (ABC kit elite, Vector, Burlingame, CA, USA), to detect the presence of T lymphocytes (expressing surface membrane CD3 marker), B lymphocytes (expressing surface membrane CD79α), and activated macrophages (expressing cytoplasmic ionized calcium binding adaptor molecule 1 (Iba1)). Normal and pathologic mesothelium were better visualized by immunolabeling with Pan-cytokeratin (AE1/AE3).

Formalin-fixed and paraffin wax-embedded tissues were sectioned (3 μm), mounted on poly-L-lysine coated slides, dewaxed, rehydrated, and rinsed with tap water at room temperature. The primary and secondary antibodies, dilutions, antigen retrieval methods, and tissues used as positive controls are reported in Table 1.

Prior to antigen retrieval, endogenous peroxidase was blocked by immersion in H_2_O_2_ 3% in methanol for 30 min. Blocking of unspecific antigenic sites was achieved by incubating the slides in a solution of 10% goat serum and PBS for 30′ at room temperature; primary and secondary antibodies were diluted in the same solution. To remove each reagent, slides were rinsed in TRIS. Slides were incubated with the primary antibody overnight at 4 °C and the reaction was visualized with 3,3′-Diamino-187 benzidine in tablets (DAB chromogen/substrate kit; Diagnostic BioSystem, Pleasanton, CA, USA), except in the case of CD3 and CD79α, which were stained with 3-amino-9-ethylcarbazole chromogen (AEC, Zymed Labs, San Francisco, CA, USA). Slides were counterstained with Mayer’s hematoxylin (Sigma Chemical Co., St. Louis, MO, USA) and permanently mounted with DPX mountant. Corresponding negative control slides were processed in parallel by replacing the primary antibody with an irrelevant, isotype-matched antibody to control for nonspecific binding of the secondary antibody. Samples were scored at the light microscope for number of cells stained. Each slide was evaluated by two pathologists (BB and ML) who reached a consensus diagnosis.

The sections were scored semi-quantitatively for each antibody at 100× magnification. Immune cells immunolabeled by CD3, CD79α and Iba1 were semiquantitatively graded on a 4-point scale (0 = no positive cells; 1 = rare positive cells; 2 = moderate number of positive cells; 3 = numerous positive cells) and scored considering location in the pericardium (visceral/superficial layer, fibrous/deep zones, and whole/total). For each marker 10 representative 400× fields of 2.37 mm^2^ diameter were evaluated.

Statistical analysis was performed with Statistica software (GraphPad Prism 9 and Statsoft). Normal distribution of the data was assessed with the Shapiro–Wilk test. Continuous variables are expressed as median, [range], mean ± standard deviation; for non-normally distributed data, the median was indicated. Correlations between the data obtained were assessed with the Spearman nonparametric test. The direct method of calculating the survival rate was applied, and the log-rank test was used to evaluate the survival variables. Normally distributed continuous data were assessed with a *t*-test. *p* < 0.05 was considered significant; *p* < 0.1 was regarded as trending towards significance.

## 3. Results

Complete data regarding this caseload are reported in Supplemental Material File S1.

### 3.1. Signalment

Twenty-seven dogs that underwent pericardiectomy, including 22 IPs and 5 PMs were included in the present study. The dogs were referred for clinical signs consistent with recurrent pericardial effusion. The cohort comprised 16 intact males, 5 intact females, 3 spayed females, and 3 castrated dogs. Intact males, comprising 59.25% of the cohort, were overrepresented.

Breeds included 6 mixed-breed dogs, 5 Golden Retriever, 4 Labrador Retriever, 4 German Shepherd, 1 English Setter, 1 Bull Terrier, 1 French Bulldog, 1 Italian Bracco, 1 poodle, 1 American Staffordshire terrier, 1 Dogue de Bordeaux, and 1 Cane Corso.

The mean age at diagnosis was 10.6 ± 2.53 years [range (7–15), median 9 years].

### 3.2. Macroscopic Examination

**Figure 1:**
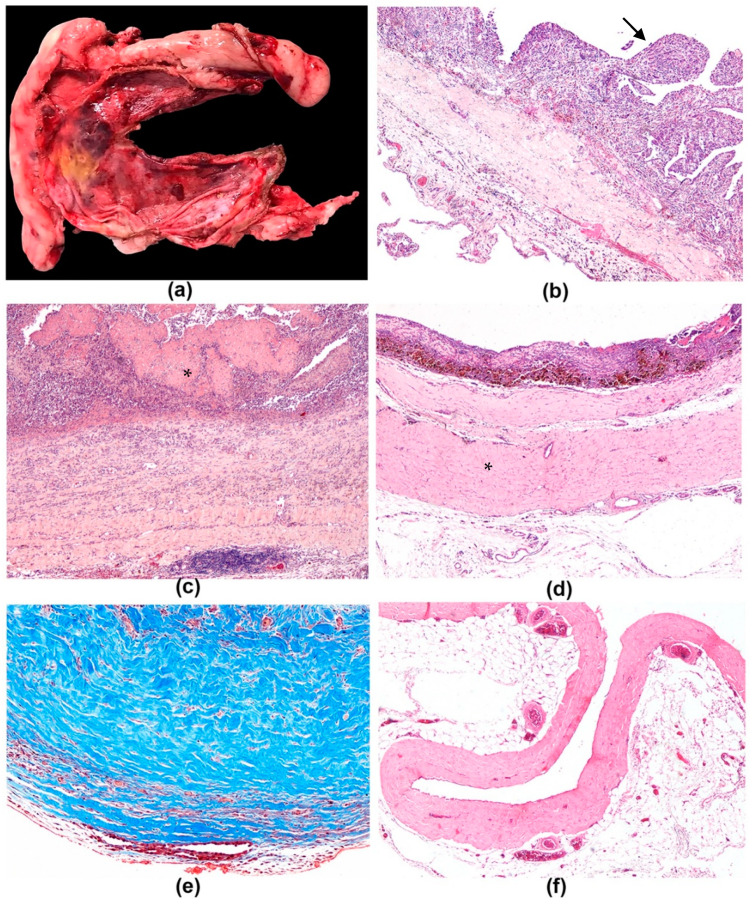
Idiopathic pericarditis, dog. (**a**) Pericardial sac of dog that underwent subtotal pericardiectomy, diagnosed with chronic idiopathic pericarditis. Serosa is variably thickened by multifocal-coalescing whitish areas of fibrosis; multifocal areas of hemorrhages and a focal area of gray-yellowish fibrin exudation at the inner/cardiac side of the serosa are present, 40×. (**b**–**d**) Idiopathic pericarditis, dog, H&E. (**b**) Mesothelial papillary hyperplasia, simple to branching papillae (arrow) lined by cuboidal mesothelial cells supported by a small axis of fibrovascular stroma infiltrated with inflammatory cells, 100×. (**c**) Fibrinous pericarditis, pericardium is covered by a thick mat of fibrin (asterisk) and expanded by abundant fibrosis in which multifocal aggregates of inflammatory cells are embedded, 100×. (**d**) Fibrous pericarditis, pericardium is replaced by abundant fibrosis (asterisk), 100×. (**e**) Idiopathic pericarditis, dog, Masson’s trichrome. Mature collagen is stained in blue, 100×. (**f**) Control pericardial tissue appeared as normal pericardium with the inner, cardiac surface covered by a single layer of flat mesothelial cells; the parietal pericardium was composed of poorly vascularized fibrous tissue and overlies mediastinal adipose tissue, 40×.

At macroscopic examination, pathological pericardial samples were opaque, wrinkled, composed of a serosal membrane variably thickened by multifocal-coalescing areas of fibrosis, and presenting multifocal areas of hemorrhages and aggregates of granulation tissue-like material in the internal/cardiac aspect (Figure 1a).

The visceral/internal/cardiac mesothelium was occasionally thickened in dense small papillary villous projections conferring a velvet-like appearance. The parietal/external/pleural mesothelial layer was unremarkable. A variable amount of adipose tissue expanded the parietal pericardium. None of the 5 pericardia diagnosed with mesothelioma presented with discrete and/or confluent macroscopic masses or plaque such as lesions on serosal surfaces that could indicate the presence of a neoplasm; therefore, differentiation between IP and PM was not feasible at macroscopic examination. The pericardial samples used as controls were thin, smooth, shiny, and transparent.

### 3.3. Microscopic Examination

IP and PM were diagnosed in 22 and 5 pericardia, respectively.

Microscopic lesions identified in IPs and PMs are presented in Table 2 and Table 3, respectively. Morphologic changes seen in pericardia of this cohort consisted of thickening of the pericardium by variable amount of fibrosis, immune cell infiltrates, hemorrhage, edema, and granulation tissue stratified at expanding the visceral/superficial and fibrous/deep zones of the pericardium. The visceral/internal/cardiac pericardial surface presented inflammatory changes involving the mesothelium and accompanied by variable extent of fibrinous exudation.

In IPs, four main mesothelial lesions were observed: The mesothelium was multifocally to diffusely lost in 20 out of 22 cases; reactive mesothelium characterized by a single layer of cuboidal cells, with sometimes distinct nucleoli, was seen in 9 out of 22 cases; MPH, characterized by simple or branching papillae lined by cuboidal mesothelial cells supported by a thin axis of fibrovascular stroma infiltrated with immune cells, was detected in 4 out of 22 cases (Figure 1b); atypical mesothelial proliferation was found in 3 out of 22 cases and was characterized by prominent mesothelial proliferation: from a single layer of cells or occasionally small tubule-like structures with more prominent atypia, to focal, raised accumulations of cells with marked anisocytosis and anisokaryosis and prominent nucleoli. In one case of IP, mesothelial cells were observed in lympho-vascular structures forming few emboli.

MPH was statistically correlated with the amount of lymphoplasmacytic infiltrate in the superficial layer (*p* = 0.01, R = 0.49), and inversely related to the thickness of the pericardium (*p* = 0.04, R = −0.43).

At histology, a mixed population of immune cell infiltrate was found with variable prevalence of neutrophils, lymphocytes, plasma cells and macrophages, sparse or in multifocal perivascular aggregates. Immune cells were stratified in a visceral/superficial zone located in the lamina propria immediately beneath the mesothelium within loose connective tissue, and a fibrous/deeper zone in the middle of the pericardium, adjacent to a layer of more densely packed collagen. Inflammation was chronic or chronic–active in all cases.

Loss of mesothelial cells was accompanied by the presence of granulation tissue in the visceral/superficial zone of the pericardium, associated with a variable number of immune cells. Abundant superficial fibrin exudation (Figure 1c) was found in a minority of cases, mostly in association with extensive mesothelial cell loss. In some cases, colorless extracellular spaces (edema) expanded the superficial pericardial interstitial space.

Extravasated erythrocytes were found both at the visceral/superficial and fibrous/deeper pericardial zones and were oftentimes associated with the presence of macrophages engulfing golden-brown hemosiderin pigment (hemosiderophages).

Thickening of the pericardium by collagen admixed with fibroblasts was the main finding in IPs and was found in variable degrees in 19 out of 22 cases (Figure 1d). The extent of fibrosis was evaluated in Masson’s trichrome stained slides (Figure 1e). The visceral/superficial pericardium was mostly characterized by deposition of loose collagen fibers associated with numerous fibroblasts with evident ovoid nucleus and occasionally evident nucleolus (reactive fibroblasts), whilst fibrosis in the fibrous/deeper zone presented denser collagen bundles admixed with enlarged fibroblasts and few newly proliferated small-sized blood capillaries. Fibrosis severity was significantly correlated to lymphoplasmacytic infiltrate in the deep zone (*p* = 0.04, R = 0.42 Spearman test).

The thickness of the pericardium, measured by image analysis and expressed as a continuous variable, was correlated with the amount of lymphoplasmacytic cells (*p* = 0.003, R = 0.60 Spearman test), and macrophages (*p* = 0.002, R = 0.61 Spearman test) in the fibrous/deep zone. The thickness of the pericardium was inversely related to MPH (*p* = 0.04, R = −0.42 Spearman test).

Neither inflammatory nor degenerative lesions affecting blood and lymphatic vessels were observed in the present study.

At histologic examination, normal pericardial tissue of control dogs was lined by a single layer of flat mesothelial cells lining the cardiac/internal surface of the serosa; the submesothelial and deep area were separated by an inconspicuous band of rare small blood vessels and the connective tissue was composed of dense collagen fibers admixed to scant flattened fibroblasts and fibrocytes; a variable amount of adipose tissue was found on the external/parietal side of the serosa which was lined by unremarkable flattened mesothelium (Figure 1f).

**Figure 2:**
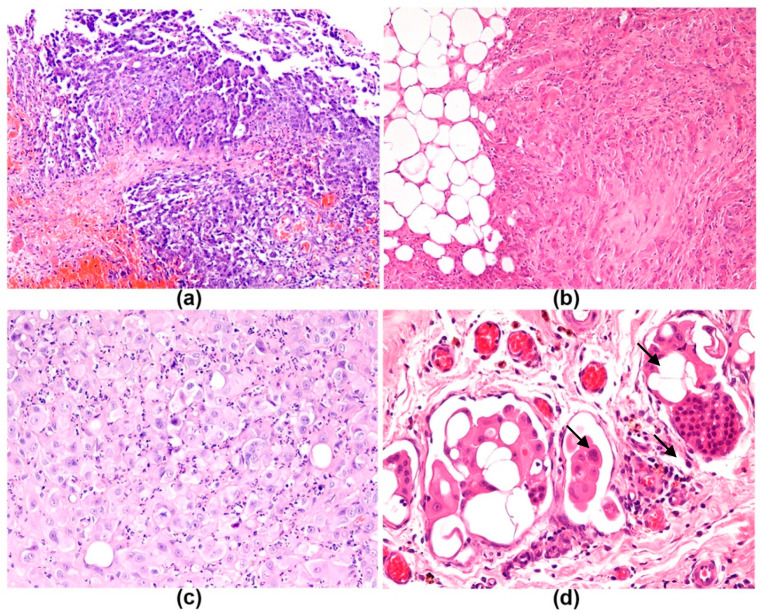
Mesothelioma, dog, H&E. (**a**) Papillary pattern of epithelioid mesothelioma. Thin papillae are visible, 100×. (**b**) Desmoplastic pattern of mesothelioma. Islands and cords of atypical polygonal cells are supported by abundant desmoplastic stroma, 200×. (**c**) Deciduoid mesothelioma. Large cells with abundant glassy eosinophilic cytoplasm, central nucleus and prominent nucleolus are present. (**d**) Solid mesothelioma. Multiple neoplastic emboli in lymphatic vessels (arrows), 400×.

Mesotheliomas were diagnosed according to the presence of a high degree of atypia and marked proliferation of neoplastic mesothelial cells, associated with aggregates of neoplastic cells invading through the lamina propria of the visceral/superficial pericardium and invading through the connective tissue in the fibrous pericardium with distortion of the normal layers. An epithelioid–papillary histologic pattern was observed in two PMs (Figure 2a), one was epithelioid–solid, one epithelioid-desmoplastic (Figure 2b), and one deciduoid (Figure 2c). Neoplastic cells were characterized by polygonal to spindle shaped cells. Foci of severe cellular pleomorphism, moderate to marked anisocytosis and anisokaryosis were consistently observed. Mitosis was 10 to 20 per 2.37 mm^2^, except in one case with 40 mitosis per 2.37 mm^2^. In two cases, neoplastic cells invasion of the serosa was transmural, reaching the adipose tissue and the parietal/outer/pleural pericardium. Lymphatic invasion by numerous pleomorphic mesothelial cells characterized by severe anisocytosis and anisokaryosis forming multiple emboli was present in 4 out of 5 PMs (Figure 2d) with massive expansion of the adipose pericardial tissue (this area was not included in the scoring). Rare focal aggregates of mesothelial cells embolized in pericardial lymphatics, observed as well in one case of IP in the present study, were not considered a criterion of malignancy as reactive mesothelial cells have been found in subserosal pericardial lymphatics [25,26]. The pericardia affected by mesotheliomas were also expanded by a mixed inflammatory infiltrate, mainly composed of macrophages at the visceral/internal/cardiac aspect of the pericardium, and by a moderate number of perivascular neutrophils and aggregates of lymphocytes and plasma cells. Edema and granulation tissue often accompanied mesothelial neoplastic proliferation (Table 3).

Comparing IP and PM cases, the histological features of edema (*p* = 0.004, R = 0.53 Spearman test), granulation tissue (*p* = 0.02, R = 0.42), macrophages in the visceral/superficial pericardial layer (*p* = 0.04 R = 0.38) and fibrin (*p* = 0.003 R = 0.53) significantly correlated with a diagnosis of mesothelioma.

Pericardial thickness measurement was the only variable with normal distribution and was significantly higher in pathologic pericardia (IPs and PMs) than in the controls (*p* < 0.0001 *t*-test). In IPs, the mean pericardial thickness was 1698 ± 559.2 μm [range (583.4–2699), median 1797 μm]. PMs had mean pericardial thickness of 1664 ± 449.1 μm [range (1203–2359), median 1505 μm]. No statistically significant difference between the thickness of IPs and PMs was found (*p* = 0.89 *t*-test).

Normal pericardia of controls had mean pericardial thickness of 171.4 ± 75.1 μm [range (108.6–254.7), median 151 μm].

### 3.4. Immunohistochemistry

Immunohistochemical scores in IPs and PMs are reported in Table 4 and Table 5, respectively.

**Figure 3:**
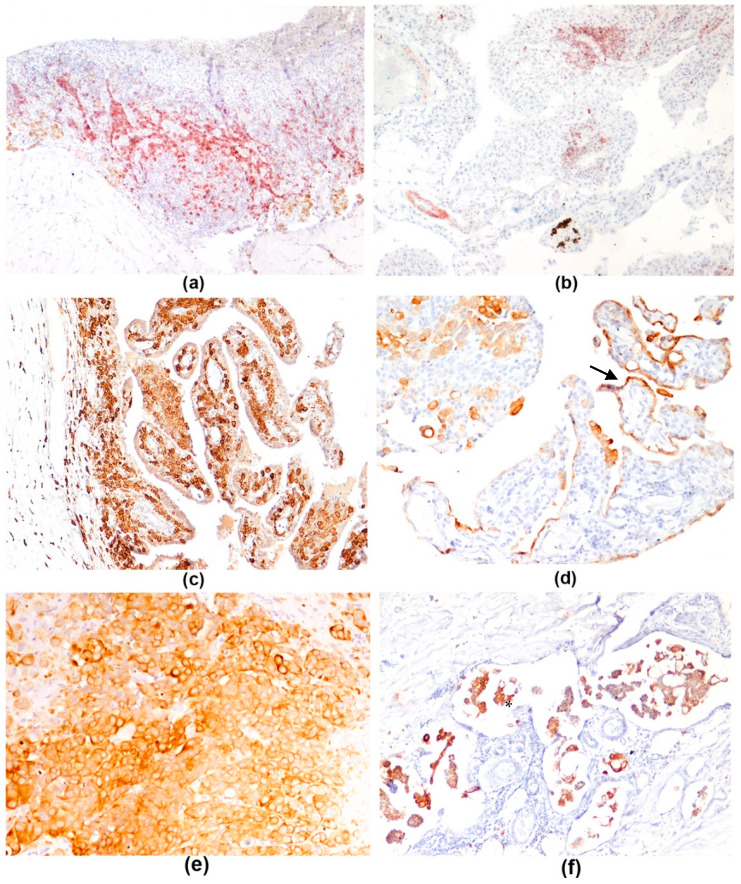
Pericardium, dog, immunohistochemistry. (**a**) Idiopathic pericarditis. Pericardium is infiltrated by numerous lymphocytes CD3 positive, stained in red (AEC), 40×. (**b**) Idiopathic pericarditis. Few lymphocytes CD79a positive, stained in red (AEC), are present in the reactive papillae, 40×. (**c**) Idiopathic pericarditis. Numerous Iba1 positive macrophages, stained in brown (DAB) are present in the papillary hyperplasia of mesothelium, 100×; (**d**) Idiopathic pericarditis. A single layer of mesothelial cells (arrow) covering hyperplastic papillae is highlighted with pan-cytokeratin, 100×; (**e**) Solid mesothelioma. Multilayering of intense pan-cytokeratin positive neoplastic mesothelial cells, stained in brown (DAB), 200×. (**f**). Solid mesothelioma. Multiple neoplastic emboli are pan-cytokeratin positive (asterisk), 200×.

CD3 membranous immunolabeling stained T lymphocytes, which were found in moderate to few numbers at the visceral/superficial aspect of the pericardium and more numerous at the fibrous/deep zone (Figure 3a) in 86% of IPs and all PMs.

B lymphocytes showed CD79α membranous immunolabeling. The tunica muscularis of arterioles showed positive immunolabeling. A few to a moderate number of B lymphocytes were found in 33% of IPs and in 20% PMs, both superficially and deep in the pericardium (Figure 3b).

One IP case in which CD3 and CD79α had no adequate staining quality (i.e., faint inconsistent immunostaining of lymphocytes) was excluded from the evaluation.

Iba1 immunolabeling was present in cytoplasm of macrophages. Iba1 positive macrophages were the most numerous immune cells and were observed mainly in the visceral/superficial/cardiac aspect of the pericardium, often infiltrating the axis of the hyperplastic papillae lined by mesothelium (Figure 3c) in 95% of IPs. Few to several Iba1-positive macrophages were found in the deep pericardial zone in all IP. The most numerous immune cells found in mesotheliomas were Iba1-positive macrophages, expanding the superficial layer of the serosa in 80% of PMs (Table 5).

Overall, numerous macrophages were found in both IPs and PMs especially at the superficial layer of the pericardium. T lymphocytes were observed in mild to moderate numbers and were more numerous than B lymphocytes in both IPs and PMs.

Pan-cytokeratin (AE1/AE3) immunostaining was useful for highlighting normal and reactive mesothelial cells (Figure 3d) and distinguishing them from macrophages and activated fibroblasts at the pericardial surface. Furthermore, pan-cytokeratin was useful to evaluate the mesothelial multilayering (Figure 3e) and mesothelial cells infiltrating the pericardial wall that occur in mesothelioma; neoplastic emboli in lymphovascular spaces were corroborated by pan-cytokeratin (Figure 3f). Pan-cytokeratin was expressed with a diffuse intense cytoplasmic staining in our caseload.

### 3.5. Clinical Outcome and Survival Analysis

The average time from initial presentation for pericardial effusion and pericardiectomy was 104.81 ± 281.1 days [range (1–1460), median 12 days]. Dogs underwent 1 to 4 pericardiocentesis before pericardiectomy. Thoracoscopic pericardial window surgery was performed in 16 dogs while 11 dogs underwent subtotal, subphrenic pericardiectomy via thoracotomy.

Four dogs were alive at time of last follow-up (24 months after surgery). Seven dogs died from extracardiac causes.

**Figure 4:**
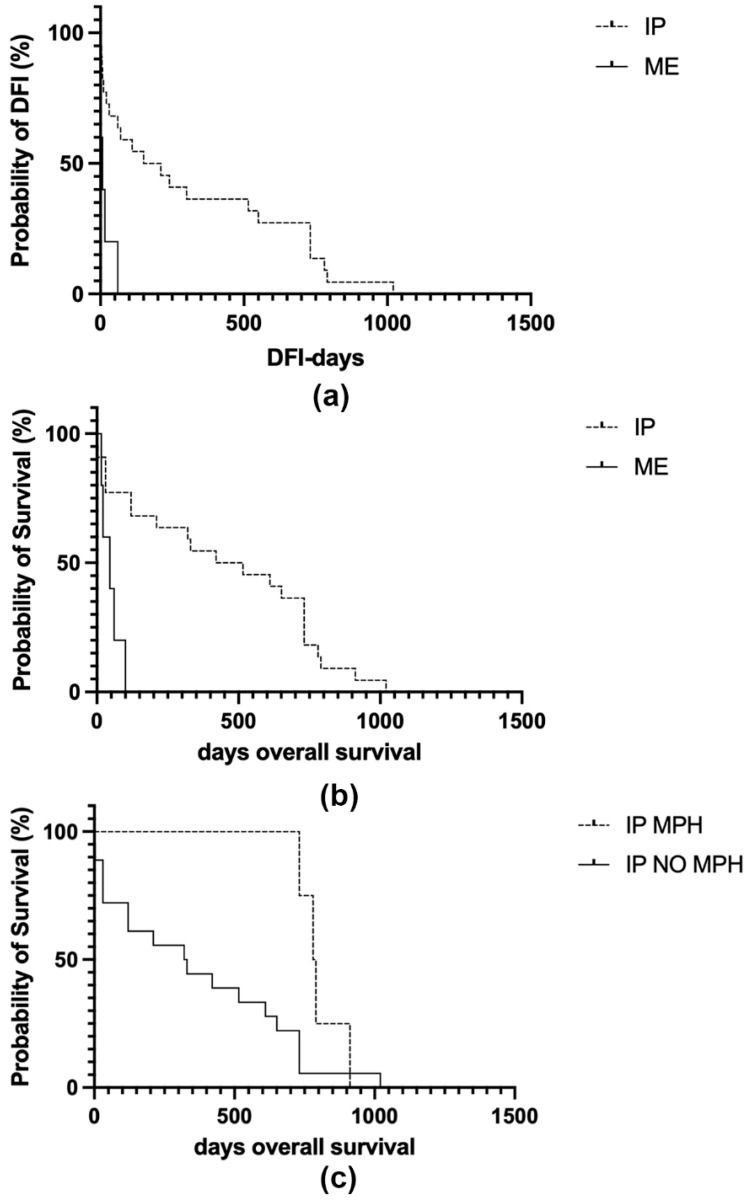
Kaplan–Meier survival curves of 22 dogs diagnosed with idiopathic pericarditis (IP) and 5 dogs with pericardial mesothelioma (ME). In each curve, the y-axis represents the survival probability. (**a**) Mesotheliomas have significantly shorter disease-free interval (DFI) times compared to IP (*p* = 0.001 log-rank test). (**b**) Mesotheliomas have significantly shorter overall survival (OS) times compared to IP (*p* = 0.0007 log-rank test). (**c**) Dogs diagnosed with IP showing the histologic feature of mesothelial papillary hyperplasia (MPH) have a significantly longer OS compared to IP cases lacking MPH (*p* = 0.04 log-rank test).

DFI of IPs was 321.1 ± 338.8 days [range (1–1020), median 180 days]. DFI of dogs diagnosed with mesothelioma was 16.8 ± 25 days [range (1–60), median 7 days]. DFI was significantly shorter in patients with a diagnosis of mesothelioma compared to those with IP (*p* = 0.002 log-rank test; Figure 4a).

OS in IP cases was 446 ± 337.1 days [range (1–1020), median 467 days]. OS and DSS coincided in dogs diagnosed with mesothelioma as they all died for euthanasia due to recurrent pleural effusion and corresponded to 48.2 ± 34.1 days [range (15–100), median 45 days]. DSS considering both IPs and PMs was 276.95 ± 285.83 days [range (1–731), median 120 days]. DSS of IP cases was 341.2 ± 295.8 days [range (1–731), median 320 days].

OS was significantly shorter in patients diagnosed with PM compared to IP (*p* = 0.001 log-rank test; Figure 4b).

A trend towards significance was found between MPH and a longer OS in patients with IP but it was not statistically associated (*p* = 0.055 log-rank test; Figure 4c) and no differences in recurrence time were observed (*p* = 0.15 log-rank test).

In IP, the semi-quantitative score of fibrosis (examined with Masson’s trichrome), was negatively correlated with DFI between 30 and 180 days (*p* = 0.04, R = −0.43 Spearman test). No statistical association between fibrosis severity, OS (*p* = 0.25 log-rank test) and DFI (*p* = 0.60 log-rank test) was detected. Time elapsed from initial presentation and pericardiectomy was correlated with the amount of fibrosis (*p* = 0.04, R = 0.39).

The number of pericardiocentesis before pericardiectomy was correlated with the diagnosis of mesothelioma (*p* = 0.04, R = 0.39) and with OS between 365 and 730 days (*p* = 0.01, R = 0.46).

## 4. Discussion

Canine IP and PM are considered elusive diseases, with little information available in recent literature [6,21,27].

The signalment data of the present caseload were consistent with literature describing idiopathic pericardial effusion as a disease of male, middle-aged, large- and giant-breed dogs [10]. Golden Retriever, German Shepherd, Great Dane, Great Pyrenean, and Saint Bernard are considered predisposed to IP development; however, only Golden Retrievers were conspicuously represented as 18.51% of the breeds in the present study. It has been hypothesized that IP is associated with the development of PM in Golden Retrievers through a chronic inflammatory process [28]; however, this was not confirmed by the data presented in this study.

Histopathologic findings, including loss and hyperplasia of mesothelium, fibrosis, and stratification of the immune cell infiltrate in both visceral/superficial and fibrous/deep zones were similarly reported in previous studies [10,12]; onion-like vascular lesions and vasculitis described by Aronsohn and colleagues (1999) and Day and colleagues (2002) were not observed in the present caseload [10,12].

No statistically significant difference between the thickness of IPs and PMs was found. In IPs, thickness was found to be lower in pericardia presenting MPH, whereas it was positively correlated with the presence of lymphoplasmacytic infiltrate and macrophages in the deep pericardial zone. A major role can therefore be attributed to immune cell infiltrates in expanding the pericardium, whilst MPH could be considered a reactive change leading to better preservation, serosal functionality and milder thickening. MPH was also found to be a potential favorable prognostic finding since it showed a trend toward significance with longer OS in IP; this positive predictive value needs to be further investigated implementing the caseload. Moreover, the thickness of the pericardium was negatively correlated with MPH, which supports a potential protective role of MHP in reducing the inflammatory reaction leading to severe thickening of the pericardium.

On the other hand, loss of mesothelium, found in 20 out of 22 cases, was commonly observed in IPs, which could lead to a leak in the function of the mesothelial barrier potentially allowing both pericardial and interstitial fluid movement across the pericardial serosa [1,4], worsening the inflammatory response and resulting in recurrent effusions. Literature on canine cardiac MPH is scarce and the pathogenesis remains unknown. Recently, Kirejezyk et al. (2018) examined 4 dogs diagnosed with acute cardiac tamponade or chronic cardiac disease and observed mesothelial cell-covered, papillary fronds from the epicardial surface of the heart. Histologically, the papillae showed variable size, were supported by an edematous fibrovascular stroma and infiltrated by variable numbers of immune cells [29]. We found a similar morphology of MPH in the pericardial internal/surface which could probably be related to fractioning between the pericardium and the beating heart covered by epicardium.

Fibrosis was found in 19 out of 22 IPs and fibrosis score was correlated with the time from initial presentation and pericardiocentesis and negatively correlated with the DFI between 30 and 180 days. Consequently, it is possible that a longer time before surgery could lead to an increased progression of the inflammation at the pericardium, resulting in more severe fibrosis compared to dogs that are treated with pericardiocentesis sooner. Deposition of a moderate to abundant amount of fibrous tissue is one of the main findings described in canine IPs [10,12]. In our study, fibrosis was correlated with the presence in the deep zone of lymphoplasmacytic infiltrates, presumably responsible for producing fibrogenic factors. Recently, in humans, various cellular events and signaling cascades have been identified, which are likely to contribute to the pathological fibrotic phenotype in constrictive pericarditis [22,30]. An initial classical pattern of inflammation arises as a result of an insult to the pericardium and can exacerbate into an exaggerated or prolonged inflammatory state [22]. However, it has been observed that about 20% of human patients with surgically proven constrictive pericarditis and focally abnormal histopathological appearance had normal pericardial thickness [31]. When comparing human and canine pericardial disease it should be borne in mind that the human pericardium is about 7 times thicker than the canine pericardium, but is more extensible in stress-strain tests, with lower stiffness at a given strain, and the human pericardium displays greater viscous responses than the canine tissue [32,33].

Overall, the immunohistochemical characterization of immune cells infiltrating the pericardium in IPs was similar to the study by Day and colleagues [12]; nonetheless, those authors found a predominance of humoral effector mechanisms (Th2 immunity), while in the present study macrophages and T lymphocytes infiltrating the pericardial wall were the most numerous immune cell component and B lymphocytes and plasma cells were a minority. The immune cellular infiltrate present in IPs examined both at histology and with immunohistochemistry confirmed the presence of an active chronic inflammation in almost all cases, characterized not only by lymphocytes, plasma cells and macrophages but also by the presence of neutrophils. The persistence of a mild to moderate number of neutrophils could have a role in the perpetuation of the inflammatory process. Nonetheless, the presence of neutrophils, particularly intravascular, might also be due to leukocyte recruitment to sites of tissue damage associated to surgical handling of the pericardium [34]. Further studies aimed to subclassify T lymphocytes (i.e., CD4 or CD8 positive) are warranted to better understand the pathogenesis of this disease.

Accurate diagnosis of mesothelial proliferations is one of pathology’s persistent challenges [35]. It is widely recognized in human and veterinary medicine that distinguishing reactive from neoplastic mesothelium can be very challenging, particularly in the pericardium as mesothelial cell reactivity is more marked in this site, presumably due to the increased local friction caused by the beating heart [18,19,24,35]. Furthermore, an association between chronic IP and development of mesothelioma has been proposed [27,28]. The challenging search for markers which can be useful in differentiating reactive from neoplastic mesothelium is still ongoing [24,36]. Currently, the diagnosis of canine PM is based on histopathological examination of H&E-stained sections of tissue obtained at surgery. However, differentiation of mesothelioma from atypical mesothelial proliferation and reactive mesothelium by IHC markers has recently been proposed to implement diagnostic precision [24]. In the present study, the benchmarks adopted for differentiating neoplastic mesothelium were those recommended in literature [6,19,24]. The main diagnostic criteria consisted of a high degree of atypia, marked proliferation of mesothelium and invasion of underlying tissues including the visceral/superficial and fibrous/deep zones of the pericardial stroma by neoplastic cells, forming cellular aggregates with evident expansion of the stroma, associated with lack of the normal layers of the pericardial wall and atypical cells within the full thickness [19]. Nonetheless, in our study, neoplastic cell aggregates were found mostly invading and distorting the visceral zone of the pericardial connective tissue, with full-thickness extension into the parietal pericardium in 2 cases out of 5. Reactive mesothelial cells entrapped in the superficial layers of the pleura can mimic invasion; however, it has been suggested that atypical mesothelial cells should not penetrate the deepest zone and infiltrate the parietal adipose tissue [18]. Mild lympho-vascular invasion by mesothelial cells was not considered as a primary feature of neoplastic lesions as it was observed in one IP case and is known to be found in inflammatory and reactive lesions of the mesothelium [25,26]. Histopathology was useful in identifying mesotheliomas based on their aspect at H&E. The morphologic criteria applied for the diagnosis of mesothelioma were useful in differentiating neoplastic mesothelial proliferation from reactive hyperplasia in IP. Mesotheliomas in this study were also found to be associated to edema, fibrin, and granulation tissue, confirming the coexistence of neoplastic and inflammatory lesions in the pericardium.

The five pericardial mesotheliomas diagnosed in the present study had a much shorter DFI than IPs (16.8 ± 25, median 7 days vs. 314.1 ± 332.7, median 160 days, respectively), and a much shorter DSS (48.2 ± 34.1 days, median 45 days vs. 341.2 ± 295.8, median 320 days, respectively). A possible association between the number of pericardiocentesis and the diagnosis of mesothelioma was observed. In fact, in the present study, dogs with PM received 3 to 4 pericardiocentesis in a minimum 5-day time frame or a maximum 53-day time frame. However, more than being a causal relationship it could be related to an increased volume and severity of the pericardial effusion rate in dogs affected by PMs requiring an increased number of drainages to alleviate the symptoms of pericardial effusion. It has been hypothesized that in the Golden Retriever mesothelioma can occur from chronic IP in dogs that underwent multiple pericardiocentesis [28]; this causality was not supported by findings in the present study, since only one Golden Retriever was diagnosed with PM in our cohort. Nonetheless, studies with a wider caseload are warranted.

DFI—defined as the time elapsed from pericardiectomy to the appearance of clinically evident pleural effusion—is the most reliable indication at follow-up for IP, as DSS and OS are subject to major influence by the compliance of owners to perform repeated thoracentesis to alleviate signs of recurrent effusions, which consequently prolong OS. On the other hand, humane euthanasia is likely to be considered earlier when owners know their animal has a diagnosis of mesothelioma thus influencing their decision-making process and OS.

Care must be taken not to over-interpret the findings since limitations of the present study include the retrospective way information was collected and the small number of cases presented. Nonetheless, this caseload is valuable since it collects IPs and PMs in association with anamnestic and follow-up data, which are often challenging to obtain. In addition, the IPs included in this caseload possibly encompass an advanced stage of this disease, since they all needed surgical treatment, being therefore not representative of early-stage canine IP. The need for validation of the present scoring system extended to wider caseload and interobserver variability should also be considered.

## 5. Conclusions

Histopathologic evaluation of pericardia in dogs that underwent pericardiectomy was useful in providing a possible prognostic indicator for IP, with MPH showing a trend toward significance with longer OS and being correlated with thinner pericardia. Fibrosis was more severe in cases with longer time from initial presentation and pericardiectomy. Pericardial mesotheliomas were identified at histopathologic examination using the criteria of invasion of pericardial serosal and fibrous stroma by neoplastic cells, with marked anisocytosis and anisokaryosis, forming cellular aggregates with distortion of the visceral/superficial zone of pericardial connective tissue. Prognosis was poorer than in dogs with IP, with a significantly shorter DFI and DSS in dogs affected by PMs, treated with pericardiectomy.

## Figures and Tables

**Table 1:** Information on immunohistochemical reagents and procedures.

Marker	Clone	Supplier	Dilution of Primary Antibody/Incubation Time	Antigen Retrieval	Positive CTR
CD3	CD3-12	Leucocyte’s antigen laboratory, UC Davis	1:20/overnight	Citrate buffer pH 6.00 in microwave at 750 W for 10′	Canine lymph node—paracortical areas
CD79α	HM57	Santa Cruz	1:200/overnight	EDTA buffer pH 8.00 in microwave at 750 W for 10′	Canine lymph node—germinal follicles
Iba1	Poly-clonal	Novus Bio	1:2000/overnight	Citrate buffer pH 6.00 in microwave at 750 W for 10′	Canine lymph node—macrophages in medullary sinuses
Pan-cytokeratin	AE1/AE3	Dako	1:300/overnight	Citrate buffer pH 6.00 in microwave at 750 W for 10′	Canine skin—epidermis

**Table 2:** Histopathologic lesions in pericardia affected by IP, evaluated on H&E sections, except fibrosis which was scored in Masson’s trichrome-stained sections.

Histopathologic Lesions	Score				-
Total N = 22	Present N (%)	Mild/1 N (%)	Moderate/2 N (%)	Severe/3 N (%)	Absent/0 N (%)
**Mesothelium**					
Loss	20 (90.9)	2 (9.9)	8 (34.8)	10 (43.2)	2 (9.8)
Reactive	9 (39)	2 (9.9)	6 (26.5)	1 (5.7)	13 (55.7)
Papillary hyperplasia	4 (18.2)	0	1 (5.7)	3 (14)	18 (76.5)
Atypical proliferation	3 (14)	0	1 (5.7)	2 (9.9)	19 (80.7)
**Superficial/visceral fibrin exudation**	7 (30.7)	5 (22.5)	1 (5.7)	1 (5.7)	15 (64)
**Edema**	4 (18.2)	2 (9.9)	2 (9.9)	0	18 (76.5)
**Hemorrhage**	14 (58.5)	6 (26.5)	4 (18.2)	4 (18.2)	8 (39.4)
**Granulation tissue**	14 (58.5)	7 (30.7)	4 (18.2)	3 (14)	8 (39.4)
**Fibrosis (Masson’s trichrome)**	19 (80.7)	2 (9.9)	11 (50)	6 (26.5)	3 (14)
**Neutrophils, infiltrate**					
Intravascular/marginating	14 (58.5)	10 (43.2)	3 (14)	1 (5.7)	8 (39.4)
Perivascular Total	16 (78.3)	12 (51.5)	0	4 (18.2)	6 (26.5)
	17 (72.4)	13 (55.7)	0	4 (18.2)	5 (22.4)
**Lymphocytes and plasma cells, infiltrate**					
Visceral/superficial zone Fibrous/deep zone Total	17 (72.4)	11 (47.4)	5 (22.4)	1 (5.7)	5 (22.4)
	19 (80.7)	12 (51.5)	2 (9.9)	5 (22.4)	3 (14)
	20 (84.9)	9 (39)	9 (39)	2 (9.9)	2 (9.9)
**Macrophages, infiltrate**					
Visceral/superficial zone Fibrous/deep zone Total	16 (68.2)	12 (51.5)	4 (18.2)	0	6 (26.5)
	20 (84.9)	12 (51.5)	7 (30.7)	1 (5.7)	2 (9.9)
	20 (84.9)	11 (47.4)	9 (39)	0	2 (9.9)
**Overall inflammation severity ^§^**	22 (100)	12 (51.5)	5 (22.4)	5 (22.4)	0

^§^ Overall inflammation severity included a score comprehensive of all inflammatory findings (fibrin exudation, edema, hemorrhage, granulation tissue, presence of infiltrates of inflammatory cells).

**Table 3:** Histopathologic lesions in pericardia affected by mesothelioma. Evaluated on H&E sections, except fibrosis which was scored in Masson’s trichrome-stained sections.

Histopathologic Lesions	Score				
Total N = 5	Present N (%)	1/Mild N (%)	2/Moderate N (%)	3/Severe N (%)	0/Absent N (%)
**Mesothelium**					
Loss Neoplastic	5 (100)	0	1 (20)	4 (80)	0
	5 (100)	NA	NA	NA	NA
**Superficial/visceral fibrin exudation**	5 (100)	3 (60)	1 (20)	1 (20)	0
**Edema**	4 (80)	2 (40)	1 (20)	1 (20)	1 (20)
**Hemorrhage**	5 (100)	1 (20)	4 (80)	0	0
**Granulation tissue**	5 (100)	0	4 (80)	1 (20)	0
**Fibrosis (Masson’s trichrome)**	5 (100)	1 (20)	3 (60)	1 (20)	0
**Neutrophils, infiltrate**					
Intravascular/marginating Perivascular Total	2 (40)	2 (40)	0	0	0
	5 (100)	2 (40)	2 (40)	1 (20)	0
	5 (100)	2 (40)	2 (40)	1 (20)	0
**Lymphocytes and plasma cells, infiltrate**					
Visceral/superficial zone Fibrous/deep zone Total	5 (100)	3 (60)	2 (40)	0	0
	5 (100)	2 (40)	2 (40)	1 (20)	0
	5 (100)	1 (20)	4 (80)	0	0
**Macrophages, infiltrate**					
Visceral/superficial zone Fibrous/deep zone Total	5 (100)	2 (40)	3 (60)	0	0
	5 (100)	3 (60)	2 (40)	0	0
	5 (100)	1 (20)	4 (80)	0	0
**Overall inflammation severity ^§^**	0	0	3 (60)	2 (40)	0

NA: not available. ^§^ Overall inflammation severity included a score comprehensive of all inflammatory findings (fibrin exudation, edema, hemorrhage, granulation tissue, presence of infiltrates of inflammatory cells).

**Table 4:** Semiquantitative scoring of IHC findings in IPs.

IHC Findings		Score				
	Total N	Present (%)	1/Mild (%)	2/Moderate (%)	3/Severe (%)	0/Absent (%)
**CD3 positive lymphocytes**						
Superficial/visceral zone Fibrous/deep zone Total	21	11 (52)	5 (24)	6 (29)	0	10 (48)
	21	14 (67)	12 (57)	2 (10)	0	7 (33)
	21	18 (86)	11 (52)	7 (33)	0	3 (14)
**CD79α positive lymphocytes**						
Superficial/visceral zone Fibrous/deep zone Total	21	3 (14)	1 (5)	2 (10)	0	18 (86)
	21	6 (29)	4 (19)	2 (10)	0	15 (71)
	21	7 (33)	6 (29)	1 (5)	0	14 (67)
**Iba1 positive macrophages**						
Superficial/visceral zone Fibrous/deep zone Total	22	21 (95)	7 (32)	5 (23)	9 (41)	1 (5)
	22	22 (100)	4 (18)	16 (73)	2 (9)	0
	22	22 (100)	3 (14)	13 (59)	6 (27)	0

**Table 5:** Semiquantitative scoring of IHC findings in pericardial mesotheliomas.

IHC Findings		Score				
	Total N = 5	Present (%)	Mild (%)	Moderate (%)	Severe (%)	Absent (%)
**CD3 positive lymphocytes**						
Superficial/visceral zone Fibrous/deep zone Total	5	3 (60)	3 (60)	0	0	2 (40)
	5	3 (60)	1 (20)	1 (20)	1 (20)	2 (40)
	5	5 (100)	4 (80)	1 (20)	0	0
**CD79α positive lymphocytes**						
Superficial/visceral zone Fibrous/deep zone Total	5	1 (20)	0	1 (20)	0	0
	5	1 (20)	0	0	1 (20)	0
	5	1 (20)	0	1 (20)	0	0
**Iba1 positive macrophages**						
Superficial/visceral zone Fibrous/deep zone Total	5	4 (80)	1 (20)	2 (40)	1 (20)	1 (20)
	5	3 (60)	1 (20)	1 (20)	1 (20)	2 (40)
	5	4 (80)	2 (40)	1 (20)	1 (20)	1 (20)

## Data Availability

The data generated or analyzed during this study are included in this published article and its supplementary information files. The raw datasets used and analyzed during the current study are available from the corresponding author on reasonable request.

## Supplementary Materials

The following are available online at https://www.mdpi.com/article/10.3390/vetsci8080162/s1, Supplementary_material_File1_Vetsci.
